# Outcomes and predictors of treatment failure following two-stage total joint arthroplasty with articulating spacers for evolutive septic arthritis

**DOI:** 10.1186/s12891-019-2652-7

**Published:** 2019-06-03

**Authors:** Chi Xu, Feng-Chih Kuo, Matthew Kheir, Xin Li, Wei Chai, Ji-Ying Chen

**Affiliations:** 1 Department of Orthopaedic Surgery, General Hospital of People’s Liberation Army, No.28 Fuxing Road, Haidian District, Beijing, 100853 China; 2 Department of Orthopaedic Surgery, Kaohsiung Chang Gung Memorial Hospital, Kaohsiung, Taiwan; 3 University of Minnesota Medical School, Minneapolis, MN USA; 4 Department of Orthopaedic Surgery, Xingtai People’s Hospital, Xingtai, Hebei Provence China

**Keywords:** Septic arthritis, Total joint arthroplasty, Erythrocyte sedimentation rate, C-reactive protein, Interleukin-6

## Abstract

**Background:**

The treatment strategy for evolutive septic arthritis (SA) with coexistent degenerative joint disease is not well established. The purposes of this study were to 1) investigate treatment outcome and potential risk factors of treatment failure in patients with evolutive SA following two-stage procedure, including insertion of an antibiotic-loaded spacer at the first stage and subsequent implantation of a new prosthesis; and 2) determine the performance of serum erythrocyte sedimentation rate (ESR), C-reactive protein (CRP), and Interleukin-6 (IL-6) in predicting persisting infection at second-stage procedure.

**Methods:**

We retrospectively reviewed 74 patients with evolutive SA of hips and knees who underwent a two-stage TJA between 2008 and 2015. The treatment success was defined according to the modified Delphi criteria and Kaplan-Meier survivorship curves were constructed to determine treatment success. A Cox regression model was performed to identify risk factors for treatment failure. Receiver operating characteristic (ROC) curves were generated to determine the prognostic value of ESR, CRP, and IL-6 in predicting persistent infection before second-stage prostheses implantation.

**Results:**

Overall, the treatment success rate was 93% for hips and 100% for knees after the first-stage surgery. The treatment success rate was 89% for hips and 84% for knees after second-stage prosthesis implantation with a mean follow-up of 4.7 (range, 2.2 to 10.8) years. Older age (Hazard ratio [HR] [per 10-year increase], 1.20; 95% confidential interval [CI], 1.11 to 1.62), higher preoperative CRP level (HR [per 1-mg/dL increase], 1.15; 95% CI, 1.04 to 1.28) and resistant organism (HR, 13.96; 95% CI, 3.29 to 19.20) were associated with an increased risk of treatment failure. All serologic tests presented limited values in predicting persisting infection, with the area under ROC curve of ESR, CRP, IL-6 and combination of the three markers was 57.8, 61.6, 60.3, and 62.1%, respectively.

**Conclusions:**

Two-stage TJA is an adequate management of infection control in patients with evolutive SA. The three potential risk factors (old age, high preoperative CRP, and resistant organism profile) may predict treatment failure following a two-stage procedure for evolutive SA. Additionally, serum ESR, CRP, and IL-6 had no benefit in predicting persisting infection before second-stage prostheses implantation. These findings may be useful when treating patients with evolutive SA.

## Background

The evolutive septic arthritis (SA) of the hip and knee in adults is a rare but dramatically disastrous disease. Evolutive SA is known to potentially cause devastating cartilage and bone damage as well as poor joint function overall [1, 2]. Recently, the incidence of SA is increasing owing to the aging population and more invasive joint procedures performed [3]. The treatment strategy for SA with coexistent degenerative joint disease is not well established. The use of a two-stage procedure, including insertion of an antibiotic-loaded spacer at the first stage followed by systemic antibiotic use and subsequent implantation of a new prosthesis, has been suggested in recent literature [4, 5]. Although the success rate is high, there is around 10–15% failure rate in these certain population undergoing two-stage procedure for evolutive SA [5–9].

Studies have attempted to evaluate factors influencing outcomes of the two-stage exchange arthroplasty for periprosthetic joint infection (PJI), including patients’ medical conditions, microbiologic results and laboratory tests [10–13]. However, the risk factors for failure following two-stage protocol for evolutive SA remains unknown, as the majority of studies mainly aimed at reporting on the treatment outcome [5–9]. Meanwhile, these studies were with small sample sizes and without standardized criteria for PJI. Most importantly, there was no standard guidance, such as serologic tests, to determine the optimal timing of the second-stage implantation while adopting two-stage procedure for evolutive SA. Therefore, it’s crucial to identify risk factors of PJI following two-stage procedure for evolutive SA and to evaluate which serologic markers can accurately rule out persistent infection at the time of second stage implantation.

The purpose of this study was to 1) determine the rate of treatment success with the two-stage procedure for evolutive SA and identify potential risk factors of treatment failure, and 2) evaluate the performance of serologic markers in predicting persisting infection at the time of the second-stage procedure.

## Methods

### Patients

After the Institutional Review Board approval, we retrospectively reviewed patients (*n* = 89) who were teated with two-stage procedures to primary total joint arthroplasty for evolutive septic hips and knees between 2008 and 2015 in our institute. Nine patients with a follow-up less than 2 years were excluded. Six patients were also excluded as they did not undergo second-stage implantation. Therefore, a total of 74 patients with 74 joints (55 hips and 19 knees) were included in the final analyses.

### Definitions of evolutive septic arthritis

The diagnosis of evolutive SA in this study was defined on the basis of one or more of the following [4, 8]: clinical signs of infection (local erythema, tenderness, effusion, limited range of motion, or the presence of a draining sinus communication with a joint), radiographic finding with loss of articular space, destruction of femoral heads or articular cartilage, laboratory serologic tests (C-reactive protein [CRP] > 10 mg/dL, erythrocyte sedimentation rate [ESR] > 30 mm/hr), purulence during operations, or positive synovial or tissue cultures at the first stage of antibiotic spacer placement.

### Two-stage protocol and postoperative treatment for evolutive septic arthritis

An institutional standard protocol of two-stage procedures was performed. During the first-stage surgery, a femoral head or knee joint resection was performed. All infected and necrotic tissue was debrided thoroughly. Three to five cultures (synovial fluid, deep tissue and bone) were obtained for culture. The joints were irrigated with 5–9 L of an antiseptic solution. An antibiotic-loaded articulating cement spacer, containing 4–6 g vancomycin and 2–4 g meropenem per 40 g bone cement (Heraeus Medical GmbH, Wehrheim/Ts., Germany) was then inserted. The combination of vancomycin and meropenem in the bone cement was utilized in accordance with our institutional infection control department, which explained that more than 90% of the organisms isolated from patients with PJI and septic arthritis were sensitive to one or both antibiotics. After the first-stage procedure, at least 6 weeks of systematic antibiotics were prescribed. The selection of systematic antibiotics was based on culture sensitivity reports and institutional guidelines with infectious disease specialists’ consultation. In patients with negative microorganisms, an empiric, broad-spectrum antibiotic therapy was applied. The timing of implantation to a total hip or total knee arthroplasty was based on the following criteria: no clinical signs of infection, a well-healed surgical wound, and gradually decreasing ESR and CRP values. All patients had a period of at least 2-week antibiotic holiday before second-stage surgery. As there was no “gold standard” in predicting persistent infection at the time of second-stage surgery, the diagnosis of persistent infection was made intraoperatively by combined consideration of lab tests, clinical and intraoperative findings. If patients had evidence of persistent infection, a repeated spacer exchange was performed. The mean interval between 1st stage and 2nd stage was 4.9 ± 3.8 months. During the second-stage procedure, the antibiotic-loaded cement spacer was removed and the prostheses were implanted followed by re-debridement and irrigation. Three to five samples of tissues for frozen sections were obtained during surgery from tissues in which infection was suspected. An antibiotic-loaded cement (Heraeus Medical GmbH, Wehrheim/Ts., Germany), containing 1 g vancomycin per 40 g bone cement was used if cemented fixation was required. Parenteral antibiotics were given postoperatively until the intraoperative cultures were negative findings.

### Data collection

The medical records were reviewed manually in detail to retrieve pertinent information, including demographic data (gender, age, body mass index [BMI], and type of joint [knees or hips]), American Society of Anesthesiologists (ASA) score, comorbidities (diabetes mellitus, rheumatoid arthritis, smoker, alcohol abuse, coronary artery disease, and pulmonary disease), the origin of SA (postoperative, hematogenous, intra-articular injection or unknown), surgical variables (previous surgical history, the numbers of prior surgical procedures and intraoperative purulence), serologic tests (serum ESR, CRP, and interleukin-6) before second-stage implantation, and the infected organisms (Table 2). Serum interleukin-6 (IL-6) was available in 49 patients because the IL-6 test was introduced in our institution since 2012. Resistant organisms were defined as methicillin-resistant *Staphylococcus aureus*, methicillin-resistant *Staphylococcus epidermidis* and vancomycin-resistant *Enterococcus*. Any clinical signs of infection, infection-related mortality, additional spacer exchanges for infection or subsequent surgical intervention after second-stage implantation were all recorded.

### Outcome measurement

The treatment success rate following first-stage surgery to an articulating cement spacer was defined as eradication of infection without any additional spacer exchanges. The treatment success following two-stage procedure to a total joint arthroplasty was defined by a modified Delphi criteria [14, 15]: (1) infection eradication characterized by a healed wound without drainage, fistula, or pain, with no recurrence of infection; (2) no occurrence of septic joint infection-related mortality; or (3) no any additional spacer exchanges and subsequent surgical intervention for infection after spacers implantation.

### Statistical analysis

All of the statistical analyses were performed with the statistical software packages R (http://www.R-project.org, The R Foundation). The clinical characteristics between groups were compared with the use of the independent t-test or Mann-Whitney test for continuous variables and the chi-square test or Fisher’s exact test for categorical variables. A Cox regression model was used to identify risk factors for treatment failure. Hazard ratios (HRs) and 95% confidence intervals (CIs) were reported. Kaplan-Meier survivorship curves were generated at the 1-year and 2-year follow-up. The log-rank test was used to evaluate the differences in survivorship between hips and knees. Receiver operating characteristic (ROC) curves were generated using Bootstrap resampling (times = 500) to determine the prognostic value of serologic tests with treatment success as an outcome measure. The area under the ROC curve (AUC) with 95% CI was calculated. A *p*-value less than 0.05 was considered statistically significant.

## Results

Table 1 showed patients characteristics and organism profiles. There were 47 males and 27 females with a mean age (standard deviation) of 49.4 ± 16.5 years in the present study. The mean BMI was 24.7 ± 4.4 kg/m^2^ (24.6 ± 4.4 kg/m^2^ for hips and 24.9 ± 4.4 kg/m^2^ for knees). The primary source of infection was postoperative open trauma (46/74, 62.2%). Eight patients had a history of intra-articular infection before the onset of infection, and 7 patients presented with a history of hematogenous infection. The etiology of infection in the other 13 patients were unknown. Coagulase negative *Staphylococcus* was the most common organism (15/74, 20.3%). Resistant organisms were found in 4 patients (5.4%) and polymicrobial organisms were identified in 6 patients (8.1%). The negative culture rate was 32.4% (24/74).

**Table 1:** Characteristic, the origin of infection and organism profile of patients who underwent two-stage total joint arthroplasty for evolutive septic arthritis

	Total (*n* = 74)	Hip (*n* = 55)	Knee (*n* = 19)
Patient characteristics			
BMI (kg/m^2^)	24.7 ± 4.4	24.6 ± 4.4	24.9 ± 4.4
Age (year)	49.4 ± 16.5	45.8 ± 16.0	59.8 ± 13.6
Male	47 (63.5%)	41 (74.5%)	6 (31.6%)
ASA score ≥ 3	17 (23.0%)	11 (20.0%)	6 (31.6%)
Origin of infection			
Postoperative	46 (62.2%)	38 (69.1%)	8 (42.1%)
Hematogenous	7 (9.46%)	5 (9.1%)	2 (10.5%)
Intra-articular injection	8 (10.8%)	3 (5.5%)	5 (26.3%)
Unknown	13 (17.6%)	9 (16.4%)	4 (21.1%)
Preoperative microorganism			
*Staphylococcus aureus*	6 (8.1%)	2 (3.6%)	4 (21.1%)
Resistant organism	4 (5.4%)	2 (3.6%)	2 (10.5%)
Coagulase negative *Staphylococcus*	15 (20.3%)	15 (27.3%)	0 (0.0%)
Gram-negative organism	7 (9.5%)	6 (10.9%)	1 (5.3%)
Other organism	12 (16.2%)	8 (14.5%)	4 (21.1%)
Polymicrobial organism	6 (8.1%)	5 (9.1%)	1 (5.3%)
Culture negative organism	24 (32.4%)	17 (30.9%)	7 (36.8%)

*BMI* Body mass index, *ASA* American Society of Anesthesiologists

**Fig. 1:**
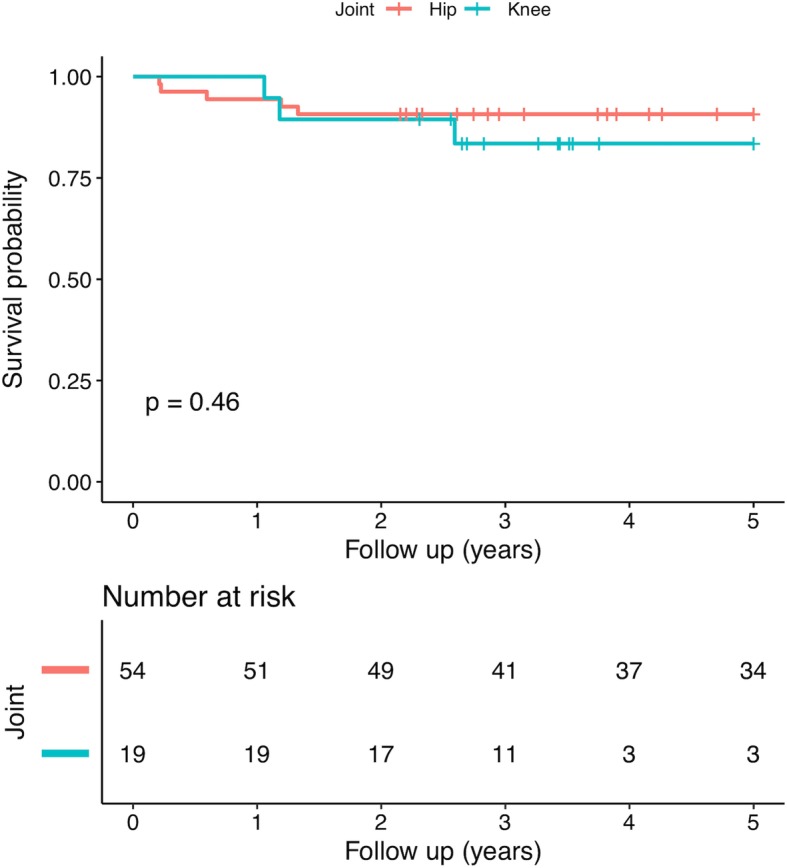
Kaplan-Meier survival curve regarding treatment failure of two-stage total joint arthroplasty for evolutive septic arthritis (SA) when stratifying by the hip and the knee

Overall, the treatment success rate was 93% for hips and 100% for knees after the first-stage surgery. The treatment success rate was 89% for hips and 84% for knees after prosthesis implantation with a mean follow-up of 4.7 (range, 2.2 to 10.8) years. There was no infection-associated mortality. The survivorship of hip with treatment success as an endpoint was 94.4% (95% CI, 88.5 to 100%) at the 1-year follow-up and 90.7% (95% CI, 83.3 to 98.8%) at the 2-year follow-up. The survivorship of knee was 94.7% (95% CI, 85.2 to 100%) at the 1-year follow-up and 89.5% (95% CI, 76.7 to 100%) at the 2-year follow-up. There was no significant difference in survivorship rate between hip and knee surgeries (*p* = 0.46) (Fig. 1). Additionally, four patients suffered from a spacer fracture and were treated conservatively. Spacer dislocation was observed in one case who was treated with conversion total hip arthroplasty. Following prosthesis implantation, eight patients had prolonged draining of the wound. One patient suffered from a prosthesis dislocation at the fourth month postoperatively and was treated by closed reduction.

The Cox regression result showed that older age (HR [per 10-year increase], 1.20; 95% CI, 1.11 to 1.62), higher preoperative CRP level (HR [per 1-mg/dL increase], 1.15; 95% CI, 1.04 to 1.28) and resistant organism (HR, 13.96; 95% CI, 3.29 to 19.20) were associated with an increased risk of treatment failure after two-stage procedure (Table 2**)**. Male (HR, 0.68; 95% CI, 0.17 to 2.80), obesity (BMI ≥30 kg/m^2^, HR, 1.11; 95% CI, 0.14 to 9.01), knee (HR, 1.43; 95% CI, 0.36 to 5.71), ASA score ≥ 3 (HR, 1.82; 95% CI, 0.75, 7.31), diabetes (HR, 2.16; 95% CI, 0.45 to 10.39), smoker (HR, 1.77; 95% CI, 0.37 to 8.52), alcohol abuse (HR, 1.90; 95% CI, 0.39 to 9.13), coronary artery disease (HR, 1.33; 95% CI, 0.17 to 10.68), pulmonary disease (HR, 3.61; 95% CI, 0.45 to 28.97), higher preoperative ESR level (HR [per 1-mm/hr. increase], 1.01; 95% CI, 0.98 to 1.05), higher IL-6 level (HR [per 1-pg/mL increase], 1.01; 95% CI, 0.97 to 1.06), surgical history (HR, 0.45; 95% CI, 0.12 to 1.67), number of surgical procedures (HR [Per-1 increase], 1.74; 95% CI, 0.67 to 49.14), intraoperative purulence (HR, 1.72; 95% CI, 0.43 to 6.87), *Staphylococcus aureus* (HR, 1.31; 95% CI, 0.16 to 10.52), coagulase negative Staphylococcus (HR, 1.31; 95% CI, 0.23 to 5.43), gram-negative organism (HR, 1.17; 95% CI, 0.15 to 9.40) and polymicrobial organism (HR, 1.47; 95% CI, 0.18 to 11.74) were not significantly associated with treatment failure.

**Table 2:** Risk factors associated with treatment failure following two-stage protocol for active septic arthritis of hips and knees

Variables	Success (*n* = 65)	Failure (*n* = 9)	HR (95% CI)	*p*-value
Patient characteristics				
Age (per 10-year increase)	48.9 ± 15.6	53.0 ± 23.3	1.20 (1.11, 1.62)	**0.021**
Male	42 (64.6%)	5 (55.6%)	0.68 (0.17, 2.80)	0.598
BMI ≥30 kg/m^2^	7 (10.9%)	1 (12.5%)	1.11 (0.14, 9.01)	0.924
Knee	16 (24.6%)	3 (33.3%)	1.43 (0.36, 5.71)	0.615
Comorbidities				
ASA ≥3	14 (21.5%)	3 (33.3%)	1.82 (0.75, 7.31)	0.186
Diabetes mellitus	7 (10.8%)	2 (22.2%)	2.16 (0.45, 10.39)	0.338
Rheumatoid arthritis	4 (6.2%)	0 (0.0%)	–	–
Smoker	9 (13.8%)	2 (22.2%)	1.77 (0.37, 8.52)	0.477
Alcohol	8 (12.3%)	2 (22.2%)	1.90 (0.39, 9.13)	0.425
Coronary artery disease	6 (9.2%)	1 (11.1%)	1.33 (0.17, 10.68)	0.787
Pulmonary disease	2 (3.1%)	1 (11.1%)	3.61 (0.45, 28.97)	0.227
Surgical variables				
Preoperative CRP (per-mg/dL)	3.4 ± 3.8	8.1 ± 10.4	1.15 (1.04, 1.28)	**0.007**
Preoperative ESR (per-mm/hr)	46.0 ± 28.2	56.4 ± 36.3	1.01 (0.98, 1.05)	0.397
IL-6 (per-pg/mL)	7.4 ± 9.0	6.6 ± 2.7	1.01 (0.97, 1.06)	0.540
Surgical history	42 (64.6%)	4 (44.4%)	0.45 (0.12, 1.67)	0.232
Prior surgical procedure(s)	0.7 ± 0.7	0.9 ± 1.4	1.74 (0.67, 49.14)	0.111
Intraoperative purulence	35 (53.8%)	6 (66.7%)	1.72 (0.43, 6.87)	0.444
Microbiology				
*Staphylococcus* aureus	5 (7.7%)	1 (11.1%)	1.31 (0.16, 10.52)	0.797
Resistant organism	1 (1.5%)	3 (33.3%)	13.96 (3.29, 19.20)	**< 0.001**
Coagulase negative *Staphylococcus*	13 (20.0%)	2 (22.2%)	1.31 (0.23, 5.43)	0.881
Gram-negative organism	6 (9.2%)	1 (11.1%)	1.17 (0.15, 9.40)	0.879
Other organism	11 (16.9%)	1 (11.1%)	0.67 (0.08, 5.37)	0.707
Polymicrobial organism	5 (7.7%)	1 (11.1%)	1.47 (0.18, 11.74)	0.718

*BMI* Body mass index, *ASA* American Society of Anesthesiologists, *ESR* Erythrocyte sedimentation rate, *CRP* C-reactive protein, *IL-6* Interleukin-6

**Fig. 2:**
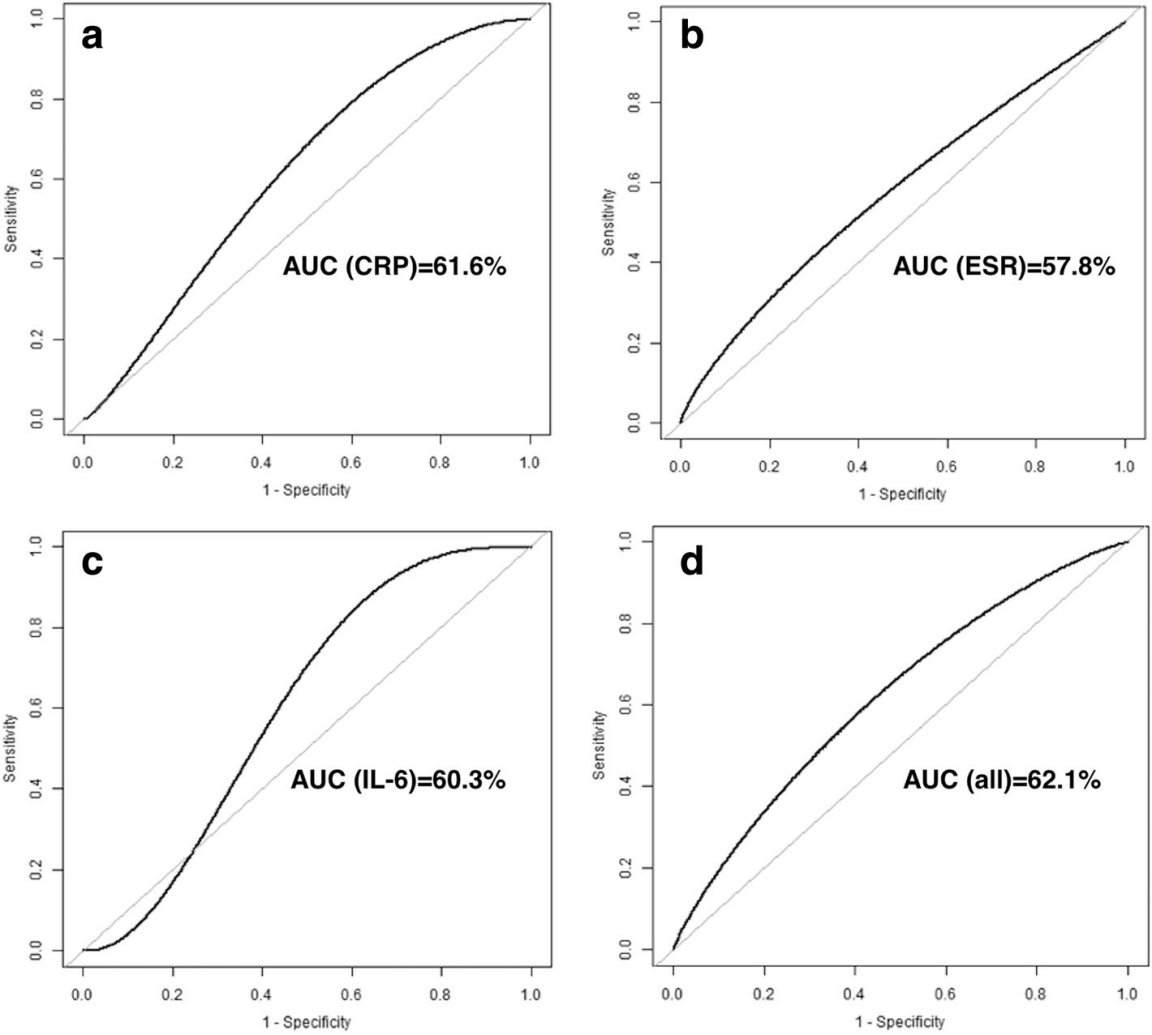
Receiver operating characteristic (ROC) curve for serum laboratory tests in diagnosing persistent infection before implantation of protheses in patients with evolutive SA: **a** C-reactive protein (CRP); **b** erythrocyte sedimentation rate (ESR); **c** interleukin-6 (IL-6); and **d** combination of the three serologic tests

The serum CRP, ESR or IL-6 values before prostheses implantation were not significantly different between treatment success and failure group (Table 3). The ROC curves for the diagnosis of persistent infection were depicted in Fig. 2. All serologic tests showed poor prognostic value in predicting persisting infection, with the AUC of 61.6% (95% CI, 42.2 to 80.2%) for CRP, 57.8% (95% CI, 37.2 to 78.8%) for ESR, 60.3% (95% CI, 43.5 to 73.3%) for IL-6, and 62.1% (42.4 to 81.0%) for the combination of the three tests.

**Table 3:** Serologic tests before prostheses implantation

Variables	Success	Failure	*p*-value
CRP (mg/dL)	0.8 ± 0.8	1.1 ± 1.0	0.134
ESR (mm/hr)	15.7 ± 18.0	19.7 ± 19.4	0.518
IL-6 (pg/mL)	8.1 ± 8.0	9.9 ± 9.8	0.404

*ESR* Erythrocyte sedimentation rate, *CRP* C-reactive protein, IL-6 Interleukin-6

## Discussion

The present study showed a satisfactory success rate of treatment with a two-stage procedure to a total joint arthroplasty (TJA) for evolutive SA of hips and knees. Older age, higher preoperative CRP level, and resistant organisms were associated with an increased risk of treatment failure. Additionally, the serological tests (ESR, CRP and IL-6) showed poor sensitivity and specificity in predicting persisting infection before second-stage prostheses implantation.

The management of evolutive SA with a coexisting degeneration joint disease remains difficult. Although prior data have reported benefits of one-stage TJA for patients with quiescent SA, there has been an increased risk of PJI following TJA [16, 17]. Furthermore, patients with active SA were contraindicated to one-stage TJA. Recently, the two-stage procedure to a TJA may be considered as a worthy alternative in the management of SA [2, 4, 5]. Papanna et al. [2] reported on 18 cases with SA who underwent one-stage or two-stage TJA based on whether the infection was active or quiescent. There was no reinfection or implant failure in this cohort at a mean follow-up of 70 months. Anagnostakos et al. [4] treated 22 patients with SA who underwent two-stage TJA. Eventually, 16 patients underwent prosthesis implantation at an average of 88 days after spacer implantation and the primary infection control rate was 87%. However, surgeons should take related complications into consideration, especially the higher mortality rate following two-stage procedures compared with other treatment methods, such as one-stage TJA. Zachary et al. conducted a meta-analysis and the pooled data suggested one-year mortality rate was 4.33% after total knee PJI with an increase of 3.13% per year mortality thereafter [18]. Another meta-analysis by Natsuhara et al. indicated one-year mortality rate was 4.22% after total hip PJI and 5-year mortality rate was 21.12%. Therefore, this highlights the importance in evaluating patients’ health conditions when determining to perform a two-stage procedure.

In our series of patients, the overall success rate was 94.6% after the first-stage surgery and 87.8% after prosthesis implantation, with a comparable incidence observed for patients with SA of the hip and knee. Although our results are similar with previous studies (Table 4), the success rates of the present study are near the lower end of rates previously reported. The reason may be that most of the studies with success rate of more than 90% included small sample sizes [23, 24]. Another explanation is that our cohort had more gram-negative infections and polymicrobial infections, which have been suggested to be associated with worse outcomes following two-stage exchange arthroplasty for PJI [25–27].

**Table 4:** Overview of current researches that reported more than five cases who underwent a two-stage procedure to a total joint arthroplasty for septic arthritis

Study	Num.	Spacer type	Antibiotic in spacer	Duration of antibiotic before reimplantation (weeks)	Period between stages (weeks)	Surgery between stages	Success rate after first-stage surgery	Duration of antibiotic after reimplantation (days)	Mean follow-up (months)	Success rate after prosthesis implantation
Hip										
Anagnostakos 2016 [4]	16	HM	GEN (1.3%) + VAN (5%)	6	13	2 SE+ 1 Girdlestone	81%	0	45	87%
Bauer 2010 [9]	13	NP	Without antibiotics	6	13	NP	NP	NP	60	85%
Chen 2008 [19]	28	Beads	OXA + GEN	> 4	15	2 Girdlestone	93%	42	77	86%
Diwanji 2008 [20]	9	HM	VAN (4.9%)	NP	23	1 SE	89%	3 to 5	42	89%
Fleck 2011 [5]	10	HM	GEN or TOB (9 to 12%) + VAN (5 to 7.5%) + Ancef (5%)	6	44	1 SE	90%	NP	28	100%
Huang 2010 [21]	14	HM	VAN (10%) + AZT (10%)	1	13	1 SE	93%	3	43	100%
Kelm 2009 [22]	8	HM	VAN (5%)	6	12	0	100%	NP	12	87.5%
Papanna 2018 [2]	11	Beads	VAN (5%)	NP	28	NP	NP	NP	70	100%
Romano 2011 [6]	19	COM	GEN (1.9%) + VAN (5%)	4	22	0	100%	28	57	95%
Shen 2013 [23]	5	HM	GEN (1.3%) + VAN (8.8%)	> 6	19	0	100%	NP	40	100%
This study	55	HM	VAN (10–15%) + MER (5–10%)	> 4	23	4 SE	93%	5	62	89%
Knee										
Bauer 2010 [9]	17	NP	Without antibiotics	6	13.3	NP	NP	NP	60	88%
Kirpalani 2005 [24]	5	Beads	NP	NP	NP	0	100%	0	38.4	100%
Nazarian 2003 [7]	14	HM	TOB (5–10%) + VAN (2.5%)	> 6	12.4	0	100%	> 185	54	100%
Shaikh 2000 [8]	13	HM	VAN (10%) + STR (5%)	> 2	22.4	1 SE	92%	> 42	48	100%
This study	19	HM	VAN (10–15%) + MER (5–10%)	> 4	20.2	0	100%	5	40.3	84%

*NP* No report, *HM* Hand-made, *COM* Commercial, *VAN* Vancomycin, *MER* Meropenem, *GEN* Gentamicin, *TOB* Tobramycin, *STR* Streptomycin, *OXA* Oxacillin, *AZT* Aztreonam, *SE* Spacer exchange

A total of 23 risk factors for potential treatment failure following two-stage TJA were investigated. Among them, only older age, higher preoperative CRP level, and resistant organisms were associated with an increased risk of treatment failure, which were in line with previous studies that evaluated risk factors for treatment failure following two-stage exchange arthroplasty for chronic PJI [10, 28, 29]. Several studies have suggested resistant organisms are associated with treatment failure after two-stage exchange arthroplasty of PJI [30, 31]. Older age was associated with gram-negative PJI and polymicrobial PJI that had higher failure rates [32, 33]. Recently, Dwyer et al. reported higher laboratory tests for diagnosis of PJI could help predict outcomes of two-stage exchange arthroplasty [11]. Additionally, other factors have been identified to associate with treatment failure among different studies. Ma et al. [30] examined 106 patients (108 knees) of PJI treated with two-stage exchange arthroplasty using 31 risk factors, and they found obesity, prolonged operative time, a history of gout and Enterococcal infection were associated with an increased risk of treatment failure. Sabry et al. [29] attempted to develop a preoperative prognostic model by evaluating patients’ individual risks for treatment failure following 314 knee PJIs that underwent two-stage exchange arthroplasty. Although the model showed satisfying results (AUC, 0.773), they were limited due to a small number of variables used in assessing treatment failure and a short follow-up. Kheir et al. [28] created a predictive calculator for surgical treatment of PJI using 1438 PJIs treated at two institutions with a total of 63 risk factors at a minimum follow-up was one year. Overall, they found ten significant risk factors for PJI treatment failure, including irrigation and debridement, history of myocardial infarction, revision surgery, presence of sinus tract, resistant organisms, ever smoker, numbers of prior surgery, synovial white blood cell count, obesity, and ESR value. However, the aforementioned models were hampered by the lack of adequately external validation.

Our results found the accuracy of serological tests, including ESR, CRP and IL-6, was poor in predicting persisting infection before second-stage prostheses implantation, which was similar with previous studies on the two-stage exchange arthroplasty of PJI. Ghanem et al. [34] reported 109 PJIs who underwent two-stage exchange arthroplasty at a single institution and suggested that ESR and CRP both had poor diagnostic performance (AUC of 0.5 and 0.54, respectively) before second-stage reimplantation. Likewise, Shukla et al. [35] reviewed 87 hip PJIs and found the poor accuracy of ESR and CRP in predicting persisting infection. A study by Kusuma et al. [36] reviewed 76 PJI cases underwent two-stage exchange arthroplasty. Although patients with infection control presented decreased ESR and CRP level before reimplantation, they failed to identify any patterns predictive of persistent infection due to their poor sensitivity and specificity. Most recently, Hoell et al. [37] reported 55 PJIs and suggested serum IL-6 was a valuable test in predicting persistent infection before reimplantation. Their results showed that the AUC of IL-6 was 0.896 and an optimal cutoff value of ≥13 pg/ml to diagnose persistent infection. However, the present study with a comparable patient sample failed to identify benefits of IL-6 in predicting persistent infection. Further studies with greater sample sizes are needed to validate these results. Additionally, some tests present promising performance in diagnosing persist infection. Several studies showed sonication of antibiotic spacers could disrupted biofilm and led to higher rates of positive intraoperative cultures [38, 39]. Kheir et al. reported that positive leukocyte esterase (LE) strip test might be used in diagnosing persistent infection and resulted in a higher rate of subsequent failure [26]. Recently, Shahi et al. reported D-dimer might be useful in diagnosing infection before reimplantation [16]. However, a meta-analysis by Lee et al. reviewed 12 studies and suggested that no single marker was superior to all the others [38]. The diagnosis of persistent infection should rely on the combination of all available tests [39].

Several limitations should be considered. Most notably, the design was retrospective and certain biases of retrospective study are inherent. For instance, types of surgery before infection and duration of infection before surgery were not available. Second, although to our best knowledge, the present study had the largest sample size in the literature, the number of patients remains small. Therefore, we didn’t separate hip and knee SA in identifying risk factors of treatment failure, which may result in under power. Third, many patients included in the present study were referred to our institution after initial management in another facility, which may result in selection bias. Fourth, although an institutional standard protocol of two-stage procedures was conducted, the treatment regime (such as antibiotic administration, the duration between first stage and second stage, antibiotic holiday before second stage) was individual. Additionally, surgeon preference of treatment regime may be a factor, which may introduce bias. Fifth, as the majority of patients with SA were treated with a two-stage procedure, we cannot compare with different treatments, such as irrigation and debridement and one-stage TJA. Further studies are needed to evaluate and compare the outcomes of different procedures. Sixth, the minimum 2-year follow-up limited to represent the long-term outcomes. Lastly, the best “cut-offs” of markers for assessment of persistent infection before second-stage surgery was not presented due to limited predictive values of these markers.

## Conclusions

Two-stage TJA is an adequate management of infection control in patients with evolutive SA. Older age, preoperative high CRP level and resistant organisms are potential risk factors of treatment failure. Additionally, serum ESR, CRP, and IL-6 had no benefit in predicting persisting infection before second-stage prostheses implantation. With the goal of optimizing risk factors and improving outcomes, further studies with larger cohorts are needed to validate these risk factors and search timely biomarkers with higher accuracies in predicting persistent infection at the time of conversion TJA.

## Data Availability

Data are available on request from the authors.

## Abbreviations

ASA: American Society of Anesthesiologists
AUC: Area under the ROC curve
CI: Confidence intervals
CRP: C-reactive protein
ESR: Serum erythrocyte sedimentation rate
HR: Hazard ratios
IL-6: Interleukin-6
PJI: Periprosthetic joint infection
ROC: Score Receiver operating characteristic
SA: Septic arthritis
TJA: Total joint arthroplasty
